# A New Departure

**Published:** 1884-01

**Authors:** 


### A New Department.

As will be seen by reference to page 47, a new department has been added to the REGISTER, which has for its editor Dr. E. G. Butty, of this city, who is well and favorably known to the readers of the REGISTER.

Dr. B. is a vigorous writer and will give *spice* and *snap* to the '*Salmagunda*" at least.

He is ready for somebody to step on his corns. But be careful.

